# Observation on prefrontal cortex activation in patients with Parkinson’s disease: a fNIRS study

**DOI:** 10.3389/fnagi.2025.1560315

**Published:** 2025-04-30

**Authors:** Yingqi Li, Tingting Hu, Yingpeng Wang, Jie Wang, Shuyan Qie, Congxiao Wang

**Affiliations:** Beijing Rehabilitation Hospital, Capital Medical University, Beijing, China

**Keywords:** Parkinson’s disease, functional near-infrared spectroscopy, prefrontal cortex, dorsolateral prefrontal cortex, dual task

## Abstract

**Background:**

Patients with Parkinson’s disease (PD) commonly experience difficulties when performing a second task while walking. The mechanisms underlying dual-task walking deficits remain poorly understood. In previous studies the second tasks were often simplistic, typically comprising questions from standardized cognitive assessments. Additionally, existing fNIRS studies comparing PD patients and healthy controls have reported inconsistent findings, limiting our understanding of prefrontal cortex (PFC) contributions to cognitive-motor integration.

**Methods:**

Forty-two healthy older adults (15 men and 27 women, age 59.97 ± 5.58 years) and fifty-eight patients with PD (25 men and 33 women, age 61.07 ± 7.56 years, Hoehn and Yahr stage 1∼3) were enrolled. The protocol consisted of three repetitions of these conditions: stationary marching and marching while two-digit arithmetic calculating. Researchers used fNIRS to measure PFC activation and changes in △HbO2 concentration during tasks execution.

**Results:**

Healthy controls demonstrated task-dependent prefrontal modulation - selective activation (6/22 channels, *p* < 0.05) during single-task conditions contrasted with global prefrontal engagement (22/22 channels, *p* < 0.05) under dual-task demands. In contrast, PD patients showed widespread activation across all 22 channel regions during both single and dual tasks (*p* < 0.05). During task switching, healthy subjects experienced significant activation increases in 15/22 channel regions (*p* < 0.05), paralleled by significant rises in ΔHbO2 concentrations across five prefrontal regions (*p* < 0.05; Cohen’s d ranging from 0.43 to 0.82). Conversely, PD patients exhibited no significant difference in the activation of all 22 channel regions (*p* > 0.05), and no significant changes of ΔHbO2 concentrations across all regions between single and dual tasks (*p* > 0.05; Cohen’s *d* < 0.30).

**Conclusion:**

Findings indicate that simple marching tasks underengage prefrontal resources in healthy individuals, whereas dual tasks engage greater prefrontal activation to meet heightened cognitive demands. In contrast, owing to disruptions in the cortico-basal ganglia-thalamocortical circuitry, PD patients exhibit a “ceiling effect” in PFC activation: increased task difficulty fails to elicit proportional activation, likely because single tasks already overtax prefrontal resources. This divergence in neural adaptability underscores core differences in cognitive-motor integration mechanisms between healthy individuals and PD patients, providing a basis for developing targeted dual-task interventions to enhance neural efficiency.

## Introduction

Parkinson’s disease (PD) is a progressive neurodegenerative disorder characterized by motor and non-motor symptoms that profoundly impact daily function (Hui et al., 2024; Lauzé et al., 2016). Dual-task walking, which involves performing concurrent cognitive or motor tasks while ambulating, is a hallmark challenge for many PD patients (such as talking or carrying a tray, which are essential for daily living) (Royall et al., 2002). Such motor-cognitive integration relies on executive function, a set of cognitive progress critical for planning, monitoring and executing a sequence of goal-directed complex actions (Royall et al., 2002). It has a significant impact on independence and therefore quality of life (QoL) (Gaßner et al., 2022; Kelly et al., 2012). This problem is common in both healthy adults and people with PD, often exacerbating disability and fall risk (Gaßner et al., 2017).

The neural mechanisms underlying dual-task walking deficits in PD patients remain poorly understood. Functional near-infrared spectroscopy (fNIRS) is a non-invasive neuroimaging technique in recent years with the advantages of being safe, non-invasive, easy to move, resistant to motion interference, immune electromagnetic interference, high temporal and spatial resolution, and capable of long-term monitoring (Wang et al., 2024). Prior fNIRS studies in healthy individuals have linked dual-task walking (e.g., walking while talking, counting, and serial subtraction) to increased PFC activation, reflecting heightened cognitive demand (George et al., 2019; Mahoney et al., 2016; Polskaia et al., 2023). However, these studies often employ overly simplistic cognitive tasks (e.g., repetitive serial subtraction like “100-7”), which are familiar to PD patients and may underestimate true cognitive load by reducing prefrontal resource engagement (Maidan et al., 2022). This methodological limitation complicates interpretation of group differences, as PD patients may require less mental effort for rehearsed tasks, potentially masking neural dysfunction.

Existing fNIRS studies comparing Parkinson’ s patients and healthy controls have yielded inconsistent findings, further complicating our understanding. Prior research demonstrated that, relative to age-matched healthy subjects, PD patients exhibit elevated PFC activition during quiet standing,. smaller relative increases in PFC activity during cognitive dual-task walking compared normal walking, and similar relative increases in PFC activition during obstacle crossing (Maidan et al., 2016). However, the efficiency of this compensatory control mechanism seems to be limited by the pool of available executive resources (Nóbrega-Sousa et al., 2020). In the study by Lin and Lin, it was observed that young adults showed reduced prefrontal activity when walking over obstacles compared to normal walking (older adults were not studied) (Lin and Lin, 2016). These discrepancies may arise from variability in cognitive task design, medication states, and prefrontal subregional coverage factors rarely systematically controlled in prior research.

Previous evidence suggests that the PFC is a critical regulator for balance and movement control (Maidan et al., 2016). In PD patients, dysfunctional basal ganglia circuits diminish motor automaticity, prompting heightened reliance on PFC-mediated cognitive control (Kelly et al., 2012; Nóbrega-Sousa et al., 2020). Concurrent research indicates that, alongside dopaminergic deficits, cholinergic system dysfunction impacts early-stage cognitive domains in PD, particularly frontal lobe executive function and attention mechanism (Maidan et al., 2016). Executive function and attention are indispensable for mobility, as cognitive control of gait and balance is fundamental to safe amubulation (Lin and Lin, 2016; Morris et al., 2019). Consequently, PFC function alterations during dual-task walking in PD patients may underlie performance deficits, warranting systematic investigation.

This study presents three key methodological advancements to complement prior research on prefrontal cortex (PFC) function in Parkinson’s disease (PD) during motor-cognitive dual tasks (Kvist et al., 2024; Maidan et al., 2016; Orcioli-Silva et al., 2020): (1) expanding neuroimaging coverage using a 22-channel fNIRS device to measure activity across six prefrontal subregions, including left lateral frontopolar cortex (lFPC), medial lateral frontopolar cortex (mFPC), right lateral frontopolar cortex (rFPC), left dorsolateral prefrontal cortex (lDLPFC), Brodmann area 8 (BA8), right dorsolateral prefrontal cortex (rDLPFC); (2) exclusively examining PD patients in the “on” medication state to isolate stable dopaminergic effects, aligning with real-world clinical scenarios and minimizing variability from fluctuating drug levels; and (3) employing intermediate-difficulty arithmetic tasks (random two-digit addition/subtraction) to engage broader PFC networks, balancing task familiarity and cognitive load to better reflect everyday challenges faced by patients. These adjustments enhanced measurement density, controlled medication state, and targeted task design allow for a more nuanced understanding of PFC subregional dynamics in PD, addressing gaps in prior work that often treated the PFC as a single unit or used overly simplistic/complex cognitive tasks.

Given that PD patients rely on prefrontal executive and attentional resources to regulate gait, assessment of PFC activity while walking is necessary to unraveling the cortical mechanisms underlying gait impairments. In this study, fNIRS was employed to measure PFC activation and changes in the concentration of oxygenated hemoglobin (HbO2) in PD patients and healthy subjects when they perform single-task marching tasks and dual-task marching cognitive tasks. By analyzing PFC activation patterns during single/dual tasks and between groups, we aimed to elucidate the cortical activation profiles and infer the neural mechanisms underlying cognitive-motor integration in PD.

## Materials and methods

### Participants

Forty-two healthy older adults (15 men and 27 women) and 58 PD patients (25 men and 33 women) were recruited from Beijing Rehabilitation Hospital. Inclusion criteria for healthy controls included: (a) 40–80 years old, (b) able to walk at least 5 min unassisted, (c) stable medications for the past month, and (d) Vital signs stable and willingness to provide informed consent and comply with the study protocol. The demographic information of the final study sample is presented in Table 1.

**TABLE 1:** General characteristics of participants.

	PD patients	Healthy subjects
Gender (male / female)	25/33	15/27
Age (years)	61.07 ± 7.56	59.97 ± 5.58
Height (m)	1.64 ± 0.07	1.64 ± 0.10
Weight (kg)	64.32 ± 7.21	65.21 ± 9.52
Course of disease (years)	6.93 ± 3.51	
H & Y (1.5 / 2 / 2.5 / 3)	12 / 23 / 11 / 12	

Inclusion criteria for PD patients were: (a) diagnosis of idiopathic PD according to the UK PD Brain Bank criteria; (b) in Hoehn and Yahr stage 1.5 3; and (c) taking anti-Parkinsonian medication. Participants were excluded if they had clinical diagnosis of dementia or other clinically significant cognitive impairment (Mini-Mental State Examination score < 24), psychiatric comorbidities (e.g., major depressive disorder), a history of clinical stroke, traumatic brain injury or other neurological disorder that could affect their performance (other than PD), any orthopedic problems that may affect their gait or had unstable medical condition, including cardiovascular instability.

All PD patients were on a stable medication regimen, and all tests were performed in the “on” state. The study was approved by the Hospital Ethics Committee, and all participants provided written informed consent.

### Experimental design

Participants were instructed to avoid strenuous activities 30 min prior to the test. Testing was conducted in a quiet private room with only the assessor and the paticipant present. Upon entering the room, participants were familiarized with the test environment and procedures, then seated comfortably for 5 min to reduce anxiety and ensure physiological stability. Subsequently, they stood unaided to don the fNIRS head cap, maintaining an upright posture with a still head throughout the protocol. Each task was performed three times across three separate blocks. Task 1 (single-task condition): participants stood quietly for 30 s as a baseline. Following a verbal cue, they performed 20 s of self-paced spot marching, followed by 20 s of quiet standing; this cycle was repeated three times (Chen et al., 2022; Kong et al., 2024; Luke et al., 2021; Rose et al., 2020). The 20 s marching task plus the 20 s standing task constitutes a block. After completing these three blocks, participants were required to stand still for 30 s. Task 2 (dual-task condition): Identical to Task 1, with the addition of concurrent two-digit arithmetic. The arithmetic tasks were presented to each participant in a randomized order generated by MATLAB scripts to minimize order-related biases.

**FIGURE 1:**
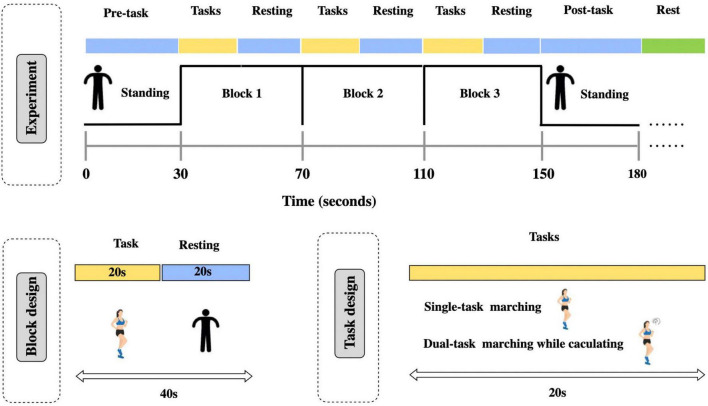
Experimental procedure.

Participants were allowed to rest between tasks until they were ready to continue. Before starting any trial, they were ensured to stand for at least 1 min to minimize blood pressure fluctuations after standing. Figure 1 illustrates the whole experimental procedure.

### fNIRS system

#### fNIRS data acquisition

**FIGURE 2:**
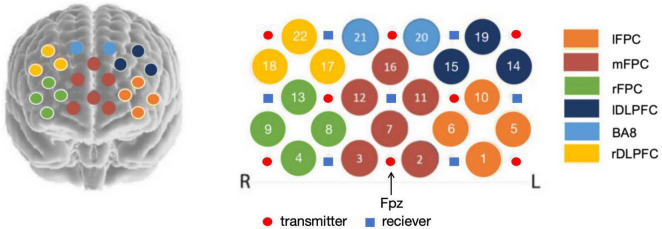
Location of FNIRS optodes, channel layout, and division of six prefrontal cortex areas. The red dot represents the transmitter, and the blue square represents the receiver. The specific channel layout of the six areas in prefrontal regions: left lateral frontopolar cortex (lFPC, channels of 1,5,6,10), medial lateral frontopolar cortex (mFPC, channels of 2,3,7,11,12,16), right lateral frontopolar cortex (rFPC, channels of 4,8,9,13), left dorsolateral prefrontal cortex (lDLPFC, channels of 14,15,19), Brodmann area 8(BA8, channels of 20.21), right dorsolateral prefrontal cortex (rDLPFC, channels of 17,18,22).

Data were collected using a 22-channel ETG-4000 NIRS device (Hitachi Medical, Tokyo, Japan) with a sampling rate of 10 Hz and a fixed distance of 3 cm between the transmitters and receiver. Based on the international 10-20 electrode system (Kruppa et al., 2021), 15 probes (8 transmitters and 7 receiver) were connected to the soft cap and arranged in a 3 × 5 grid, covering the prefrontal cortex of the subject’s brain. As illustrated in Figure 2, probe positions were anchored to the midline nasion-inion axis, with the central transmitter in the bottom row aligned to the Fpz landmark (midline frontal pole). After debugging the probe and verifying stable signal connections across all channels, the formal test commenced. Referencing the anatomical parcellation of Morris et al. (2016), the PFC was divided into 6 subregions according to Brodmann areas (BAs), with channel assignments specified in Figure 2 (McArdle et al., 2017). Neuronal activity can be measured by using the modified Beer-Lambert law to convert absorbance differences into relative concentration changes of oxygenated hemoglobin (HbO2), deoxygenated hemoglobin (HbR), and total hemoglobin (HbT) (Friedman and Robbins, 2022). Due to their superior sensitivity to regional cerebral blood flow in fNIRS and strong positive correlation with the blood-oxygen-level-dependent (BOLD) signal used in fMRI (Chen et al., 2022).

#### fNIRS data processing and analysis

The fNIRS data were preprocessed using the NIRS_KIT software package (version 3.0) under MATLAB R2020b. During data preparation, the modified Beer–Lambert law was applied to convert the original light intensity data of each channel into the relative concentration changes of HbO2 and HbR. In the first step of data processing, detrending was carried out to eliminate the influence of baseline drift. In the second step, the time derivative distributed repair (TDDR) method was used to perform motion correction on the fNIRS data, thereby removing the impact of head movement artifacts. The third step involved using an IIR band-pass filter (with a frequency range of 0.01–0.1 Hz) for filtering to eliminate the influence of physiological and non-physical noises such as respiration and heartbeat. For individual-level statistical analysis, the General Linear Model (GLM) was used to analyze how task stimuli relate to neural activation reflected in fNIRS signals. The GLM models each channel’s time-series data as a linear combination of task predictors (e.g., when a task starts/ends, encoded as stepwise signals) and a hemodynamic response function (HRF), which captures the delayed oxygenation changes caused by brain activity. The model equation is:


Y⁢(t)=∑k=1nβk•(Xk⁢(t)⁢h*⁢(t))+ϵ⁢(t)

Here, β_k_ (β-values) represent the strength of the neural response to the k-th task condition—larger values indicate stronger activation. These parameters were estimated using least squares fitting to match the model’s predictions to the actual data, minimizing errors (noise ϵ(t)). After estimating β-values, linear contrasts (e.g., single task vs. baseline) were constructed and tested via t-statistics to identify significant task effects.


$$t=\frac{c^{⊤}\,\hat{\beta }}{\sqrt{c^{⊤}\,\mathrm{Var}\,\left(\hat{\beta }\right)\,c}}$$

where c is a contrast vector (e.g., [1, −1] for pairwise comparisons). Channels with *t*-values exceeding a significance threshold (e.g., *p* < 0.05, corrected for multiple comparisons) were deemed task-responsive.

The GLM allows isolating task-related signals from noise by mathematically separating the effects of different tasks, providing a statistical framework to identify which brain regions (channels) responded significantly to specific tasks. This approach is widely used in neuroimaging for its ability to link experimental designs to neural responses, forming the basis for both individual and group-level analyses.

To compare the activity of different areas in the prefrontal cortex under different tasks, the time series of HbO2 concentration changes in each channel after preprocessing was extracted. First, the baseline value was subtracted, and then the values were averaged according to the areas where the channel is located to represent the time series of △HbO2 concentration changes in each areas. Subsequently, the average value of each area during task time series was calculated to reflect the areal activity under the task state for further statistical analysis.

### Statistical analysis

Statistical analyses were conducted using IBM SPSS Statistics 25.0 and MATLAB R2020b. One-sample t-tests were conducted on β-values to assess group-level task-evoked activation within each task and channel. The differences in activation between patients with PD and healthy subjects were calculated by two-sample t-test. The differences in activation between single task and dual task were calculated by paired-sample *t*-test. A *p*-value < 0.05 indicates that the difference is statistically significant. The statistical tests were corrected for multiple comparisons using Benjamini-Hochberg correction to control the false discovery rate (FDR). Generalized linear mixed models (GLMMs) were used to compare the concentration of △HbO2 in six prefrontal subregions among different groups and tasks. The fixed effects were group (patients with PD vs healthy subjects), task (single task vs dual task) and an interaction between group and task, with a random effect for participant. Restricted maximum likelihood estimation (REML) was employed to estimate the parameters of the GLMMs. Subsequently, pairwise comparisons were carried out. The least significant difference (LSD) method was applied to correct for Type I errors in these pairwise comparisons. Cohen’s d was calculated for each pairwise comparison to quantify the effect size, thereby offering a more detailed and nuanced understanding of the disparities in the concentration of △HbO2.

## Results

1. Prefrontal regions activation

**FIGURE 3:**
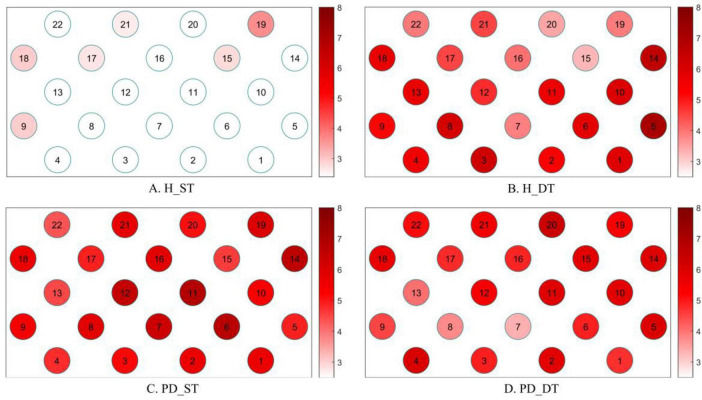
Activation status of each channel region when healthy subjects and patients with PD performing single and dual tasks, darker colors represent higher levels of activation. **(A)** H_ST, Prefrontal cortex activation in healthy people during single-task performance. **(B)** H_DT, Prefrontal cortex activation in healthy people during dual-task performance. **(C)** PD_ST, Prefrontal cortex activation in PD patients during single-task performance. **(D)** PD_DT, Prefrontal cortex activation in PD patients during dual-task performance.

When healthy controls performed a single task, only six channels (channels of 9, 15, 17, 18, and 19) among the 22 channel regions were activated (*p* < 0.05, Figure 3A). However, when they performed a dual task, all of the 22 channel regions were activated (*p* < 0.05, Figure 3B). As for patients with PD, all of the 22 channel regions were activated whether they were performing a single task or a dual task (*p* < 0.05, Figures 3C, D).

2. Differences in prefrontal region activation

**FIGURE 4:**
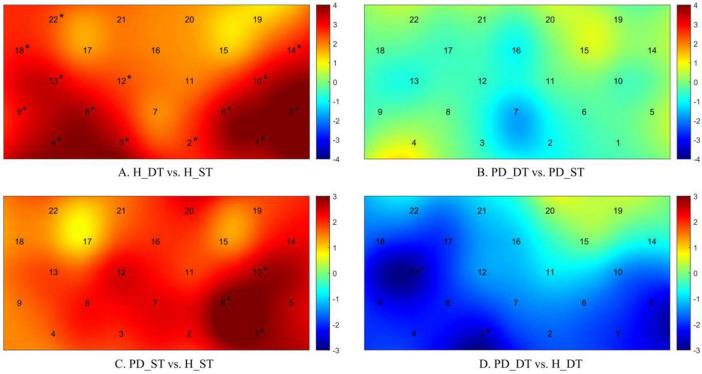
Differences in the activation of each channel regions between healthy subjects and patients with PD when performing single tasks and dual tasks. The closer the color in the image is to dark red, the more obvious the difference between the two groups is, and the opposite is true the closer the color is to dark blue. **(A)** H_DT vs. H_ST, differences in prefrontal activation when healthy people perform single and dual tasks; **(B)** PD_DT vs. PD_ST, differences in prefrontal activation when PD patients perform single and dual tasks; **(C)** PD_ST vs. H_ST, differences in prefrontal activation between PD patients and healthy people when perform single task; **(D)** PD_DT vs. H_DT, differences in prefrontal activation between PD patients and healthy people when perform dual task; *p < 0.05, there is a significant difference between the two groups.

Compared with performing a single task, most of the channel regions (channels of 1, 2, 3, 4, 5, 8, 9, 10, 11, 12, 13, 14, 16, 18, 22) of healthy subjects were significantly activated when performing a dual task, as shown in Figure 4A. By contrast, for patients with PD, there was no significant difference in the activation of all channel regions when performing a dual task compared with that when performing a single task, as shown in Figure 4B.

When performing a single task, the channel regions of 1, 6, and 10 in patients with PD were significantly activated (*p* < 0.05) compared to the healthy subjects, as shown in the Figure 4C. While performing a dual task, the activation of the channel regions of 3 and 13 in patients with PD was significantly lower than that of healthy subjects (*p* < 0.05), as shown in Figure 4D.

3. Differences in the concentrations of △HbO2 in 6 areas of the prefrontal regions

**FIGURE 5:**
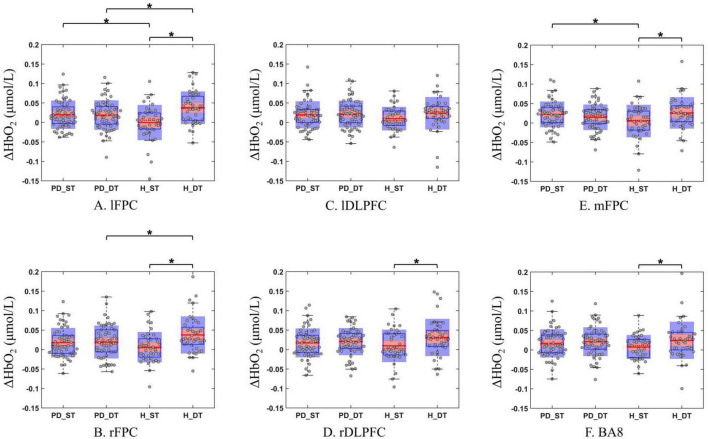
Differences in the concentrations of △HbO2 in 6 areas of the prefrontal regions between healthy subjects and patients with PD when performing single tasks and dual tasks. (**A–F**) Left lateral frontopolar cortex (lFPC), medial lateral frontopolar cortex (mFPC), right lateral frontopolar cortex (rFPC), left dorsolateral prefrontal cortex (lDLPFC), Brodmann area 8(BA8), right dorsolateral prefrontal cortex (rDLPFC). △HbO2, oxygenated hemoglobin concentration difference; PD_ST, PD patients perform single tasks; PD_DT, PD patients perform dual tasks; H_ST, healthy people perform single tasks; H_DT, healthy people perform dual tasks; *p < 0.05, difference in △HbO2 between PD patients and healthy people in a single area when performing single and dual tasks.

When healthy subjects performed single and dual tasks, significant task - related differences (*p* < 0.05) were observed in △HbO2 concentrations across five prefrontal regions (lFPC, mFPC, rFPC, BA8, and rDLPFC), and during dual tasks, higher concentrations of △HbO2 were shown (*p* < 0.05, Cohen’s d ranging from 0.43 to 0.82, Figures 5A, B, D–F and Table 2). The lDLPFC showed no significant difference (*p* = 0.062, Cohen’s *d* = 0.34, Figure 5C and Table 2). However, there was no significant difference in the concentrations of △HbO2 in all areas when patients with PD performed single and dual tasks (*p* > 0.05, Cohen’s *d* < 0.30, Figures 5A–F and Table 2).

**TABLE 2:** Statistical and effect size summary of △HbO2 differences in 6 areas of the prefrontal regions.

Region	Comparison	Notation	Mean difference (μmol/L)	Effect size (Cohen’s *d*)	*p*-value	95% confidence interval
lFPC	Healthy within-group	H_DT vs. H_ST	0.032	0.82	<0.001*	[+0.016, +0.048]
	Between-group (single task)	PD_ST vs. H_ST	−0.059	−0.62	0.001*	[−0.093, −0.025]
	Between-group (dual task)	PD_DT vs. H_DT	0.038	0.48	0.015*	[+0.007, +0.069]
mFPC	Healthy within-group	H_DT vs. H_ST	0.027	0.73	<0.001*	[+0.010, +0.044]
	Between-group (single task)	PD_ST vs. H_ST	−0.046	−0.54	0.005*	[−0.078, −0.013]
rFPC	Healthy within-group	H_DT vs. H_ST	0.022	0.61	0.015*	[+0.007, +0.038]
	Between-group (dual task)	PD_DT vs. H_DT	0.022	0.41	0.010*	[+0.005, +0.038]
BA8	Healthy within-group	H_DT vs. H_ST	0.021	0.51	0.023*	[+0.003, +0.039]
rDLPFC	Healthy within-group	H_DT vs. H_ST	0.019	0.43	0.040*	[+0.000, +0.038]
lDLPFC	Healthy within-group	H_DT vs. H_ST	0.009	0.34	0.062	[−0.001, +0.038]
All PD regions	PD within-group	PD_DT vs. PD_ST	−0.007 to +0.018	<0.30	>0.05	/

PD_ST, Parkinson’s disease (PD) single task (*n* = 58); PD_DT, PD dual task (*n* = 58); H_ST, Healthy single task (*n* = 40); H_DT, Healthy dual task (*n* = 40); Within-group: Dual task (DT) minus single task (ST); Between-group (PD-H): PD group minus healthy group (positive = PD > Healthy, negative = PD < Healthy). Within-group: Paired d = mean difference / standard deviation of differences (healthy group data). Between-group: Independent d = mean difference / pooled standard deviation.

*p < 0.05.

When performing a single task, the concentrations of △HbO2 in the lFPC and mFPC areas of patients with PD were significantly higher than that of healthy subjects (*p* < 0.05, Cohen’s d ranging from −0.62 to −0.54, Figures 5A, E and Table 2). While when performing dual tasks, the concentrations of △HbO2 in the lFPC and rFPC areas of patients with PD were significantly lower than that of healthy subjects (*p* < 0.05, Cohen’s d ranging from 0.41 to 0.48, Figures 5A, B and Table 2).

## Discussion

This study characterized PFC activation patterns in healthy subjects and PD patients during single and dual tasks, revealing a fundamental contrast in neural adaptability. Specifically, healthy subjects exhibited selective PFC engagement during single motor tasks (e.g., marching) - primarily in lDLPFC and rDLPFC, but engaged widespread PFC regions during dual tasks (marching + arithmetic), reflecting dynamic resource recruitment to meet increased cognitive demands. In sharp contrast, PD patients displayed consistent, non-adaptive activation across all 22 PFC channels in both task conditions, with no incremental recruitment during dual tasks, indicative of a fixed neural response to task demands. Group comparisons revealed task-dependent disparities: PD patients displayed hyper-activation in lFPC and mFPC subregions during single tasks, likely compensating for motor deficits, yet hypo-activation in lFPC and rFPC during dual tasks, suggesting impaired capacity for high-order cognitive-motor integration. These findings highlight a core deficit in PD patients: unlike healthy individuals, who flexibly mobilize PFC resources as task demands rise, PD patients appear to operate at a prefrontal activation threshold even during simple tasks, limiting adaptability during dual-task challenges.

During single-task performance, PD patients exhibited elevated activation across multiple prefrontal subregions compared to healthy controls. PFC is associated with cognitive control and executive function (Yu and Lui, 2022). Anatomically, the PFC communicates with the motor cortex via descending fibers, mediating critical functions such as motor planning, coordination, and execution to ensure smooth movement trajectories (Draheim et al., 2022). Additionally, it facilitates adaptive behavior by selecting context-appropriate actions and strategies, while regulating motor flexibility to accommodate novel task demands or environmental changes (Baldauf and Desimone, 2014; Morris et al., 2016). In healthy individuals, single-task stepping activated only focal PFC regions, likely reflecting automated motor control, a state where well-learned movements require minimal cognitive engagement. By contrast, PD patients displayed widespread PFC activation during simple stepping, presumably due to impaired motor automaticity: dopamine deficiency disrupts basal ganglia-mediated procedural learning, forcing reliance on explicit cognitive strategies and heightened sensory integration to execute otherwise routine motor tasks (Sarapata et al., 2023). This excessive recruitment of prefrontal resources may serve as a compensatory mechanism, where cognitive control networks supplement damaged motor circuits to maintain basic stepping competence Moreover, the cortex-basal ganglia-thalamus-cortex, as an extrapyramidal system loop, is connected to different cerebral cortical areas through the basal ganglia and thalamus respectively (Catani, 2019; Maidan et al., 2016). In PD patients, dopaminergic degeneration disrupts this circuit’s functional balance, leading to cascading cortical changes that include hyperexcitability in prefrontal motor-planning regions (Silva-Batista et al., 2024). Thus, even when PD patients perform simple marching tasks, motor deficits drive hyperactivation of the PFC, as the brain upregulates cognitive-motor integration networks to compensate for impaired subcortical control.

When performing dual tasks, the addition of calculation tasks increased cognitive load recruitment, induced significantly greater PFC activation than the single-marching task in healthy controls. This results aligns the findings of previous study by Kvist et al. (2024) DLPFC activity increased from single-task to dual-task walking in the younger group, and DLPFC activity increased from rest to single-task walking in the older and PD groups. This divergence likely stems from a “ceiling effect” in PD: single-task demands may already tax prefrontal resources to near-maximal capacity, precluding additional recruitment during dual tasks. Due to the absence of formal task-related metrics (e.g., arithmetic accuracy, marching rhythm consistency), our ability to definitively determine whether single tasks demand near-maximal PFC engagement in PD patients remains constrained. Prior research in aging and clinical populations has noted that when single-task demands approach an individual’s functional threshold, dual-tasking may not trigger additional neural activation, possibly due to prefrontal resources already being engaged at near-maximal levels during single-task performance (Wong et al., 2015).

Our findings converge with neuroimaging literature on cognitive-motor integration (Lindh-Rengifo et al., 2021; Maidan et al., 2016). A study using fMRI found that healthy subjects had greater activation in the prefrontal cortex, sensorimotor areas, and cerebellum when performing a dual-task of ankle plantar flexion and dorsiflexion and a continuous subtraction of 100 minus 3 than when performing a single task of ankle plantar flexion and dorsiflexion (Sarasso et al., 2021). This study supports our result that healthy subjects activate more brain regions during dual tasks requiring coordination. Whereas PD patients exhibit blunted responses to incremental load, likely due to fixed resource limitations. Prior fNIRS studies have demonstrated significant group differences in prefrontal HbO2 concentrations during routine tasks between PD patients and healthy people. A notable investigation using N-back memory tasks, a paradigm widely used to assess working memory load, observed nonlinear frontal lobe activation patterns: activation intensity increased with task difficulty but plateaued at high loads, reflecting a ceiling effect in neural recruitment (Linqiong et al., 2022). Specifically, the bilateral prefrontal cortex exhibited stronger activation during 2-back compared to 1-back tasks, yet no significant differences emerged between 3-back and 2-back conditions, suggesting limited capacity for further neural engagement at maximal difficulty levels (Linqiong et al., 2022). This pattern is consistent with our results that PD patients exhibit earlier attainment of PFC activation thresholds, thereby limiting additional neural recruitment during dual-task challenges. Theoretical frameworks propose that once cortical resources reach a saturation point that observed in tasks requiring escalating cognitive demand the brain cannot mobilize further neural outputs, even when task difficulty increases (Mandrick et al., 2013). Therefore, we hypothesize that PD patients exhibit maximal PFC activation during single-task performance, leaving no reserve capacity to recruit additional prefrontal regions for more complex tasks. In dual tasks, healthy subjects need more participation of the frontal cortex to perform more complex tasks than “automated tasks”, so the degree of activation of the PFC is higher than that of single tasks.

Group-level analysis of average ΔHbO2 concentrations across six prefrontal subregions mirrored the above findings: in healthy subjects, most measured regions exhibited significantly higher ΔHbO2 during dual-task than single-task performance, with the exception of lDLPFC. Neuroimaging research has found that the dorsal lateral prefrontal cortex plays a role in goal-driven attention, task switching, planning, problem-solving, and novelty-seeking (Jones and Graff-Radford, 2021). The above conclusions were also observed in our study. When healthy subjects performed a single task, channels 15, 17, 18, and 19 representing the IDPFC and rDPFC were activated, while other areas were not activated, as shown in Figure 5A. It indicated early engagement of DLPFC circuits even during simple tasks. However, there were no significant differences in the concentrations of △HbO2 values across the six subregions in PD patients when performing single and dual tasks. The concentrations of △HbO2 in the left frontopolar cortex (lFPC) region of PD patients was significantly higher than that of healthy subjects when performing a single task, but was significantly lower than that of healthy subjects when performing a dual task. Several studies using fMRI have shown that the lFPC plays a role in attention switching. The IFPC is activated when the subject reallocates attentional resources (Yahya, 2020). In this study, when PD patients performed a single task, the LFPC was more activated, reflecting that even simple marching task demand substantial attention resources. When introduced a more difficult calculation task, subjects had to complete calculation tasks while stepping simultaneously, which required relatively high attention, resulting in a relatively high degree of activation of the left frontopolar cortex. At this time, the dual marching-calculation task required significantly more attention resources for healthy subjects than single task. However, in patients with PD, the LFPC may no longer be activated to a higher degree, possibly due to damage to the underlying neural circuits.

Our study explicitly controlled for dopaminergic medication effects by testing PD patients in the “on” state (post-levodopa administration), a condition associated with optimized motor and cognitive function. This finding aligns with the study of Orcioli-Silva et al. (2020) that levodopa enhances DLPFC activation during dual tasks that critical for task coordination. While our PD patient cohort also showed widespread activation of the PFC during the “on” state (consistent with preserved dopaminergic modulation), a key difference emerged: unlike the increased dual-task activation observed by Orcioli-Silva et al. (2020) our PD patients did not show significant PFC recruitment from single to dual tasks despite a stable medication regimen. This difference could be attributed to different task demands (their study used walking-fluency tasks while ours used stationary marching + arithmetic) or a ceiling effect in our cohort, where motor-cognitive integration may have exhausted prefrontal resources even during the “on” state.

Our findings may offer practical implications for PD management. Firstly, targeted dual - task training appears beneficial. By capitalizing on residual dopaminergic function during the “on” state, such training could potentially enhance prefrontal flexibility in PD patients. Future interventions might focus on low to moderate cognitive loads, enabling patients to practice resource reallocation without overtaxing prefrontal networks. Combining fNIRS with behavioral metrics, like gait stability during dual tasks, could offer valuable insights. It may help us better understand the relationship between prefrontal cortex activation and real-world functional performance, thereby guiding more effective rehabilitation strategies. Given that gait dysfunction in PD is rooted in sensorimotor-cognitive integration deficits, hierarchical task training, neurofeedback-based interventions, and cognitive enhancement strategies may hold promise. These approaches may help overcome resource limitations within the executive-attentional network. Although further research is needed to determine their long term efficacy, they potentially optimize cortical plasticity and enhance functional independence. Overall, while more work is required to validate these ideas, our findings could contribute to the development of novel PD rehabilitation paradigms.

One limitation of this study is the small sample size. Although the sample size was sufficient to observe the differences in the prefrontal activation and the concentrations of △HbO2 between the two groups, more subjects need to be included to investigate the regular changes in activation of each brain region after more detailed functional division. In addition, unfortunately, we did not record the accuracy of the subjects’ calculation questions, so some subjects may not have performed the calculations carefully. The dual tasks we designed were relatively complex, but we did not include a simpler dual task for comparison. In addition, we cannot rule out that the marching task is already a difficult task for Parkinson’s patients, so the prefrontal cognitive area was fully mobilized and activated, resulting in a ceiling effect. Moreover, we acknowledge that this design does not account for interindividual variability in medication response (e.g., differences in absorption, metabolism, or receptor sensitivity) or compare “on” vs. “off” states. While we recorded basic medication details (e.g., levodopa dose, duration of therapy), we did not formally analyze these factors as covariates. Meanwhile, the dual tasks we designed were the stepping task and two-digit addition and subtraction, and although all the subjects had no mental arithmetic experience, we did not consider the impact of their education level on the results. Another point we did not take into account is the correlation between the subjects’ behavioral performance and prefrontal lobe activation. We will correct these shortcomings in subsequent studies and collect more information on the subjects’ performance in various aspects in order to further improve our research.

## Conclusion

This research reveals distinct PFC activation patterns during single and dual tasks in healthy individuals and PD. Healthy subjects engage limited PFC regions related to cognitive control during simple walking, with marked increases in PFC activation during dual tasks, reflecting dynamic resource allocation to meet heightened demands. PD patients exhibit a divergent strategy: lFPC and mFPC hyperactivation during single-task, yet lFPC and rPFC hypoactivation during dual-task. This dissociation indicates that even basic tasks exhaust prefrontal resources in PD, likely due to cortico-basal ganglia circuit dysfunction, thereby restricting the capacity to recruit additional neural resources for complex tasks. These findings advance understanding of cognitive-motor integration deficits in PD and inform targeted dual-task training to optimize prefrontal neural adaptability.

## Data Availability

The raw data supporting the conclusions of this article will be made available by the authors, without undue reservation.
